# The Role of Neutrophil-to-Lymphocyte Ratio and Mean Platelet Volume in Diagnosing Odontogenic and Non-odontogenic Head and Neck Abscesses

**DOI:** 10.7759/cureus.72711

**Published:** 2024-10-30

**Authors:** Gergana M Chausheva, Yanko G Yankov, Diana D Nenova

**Affiliations:** 1 Department of Central Clinical Laboratory, University Hospital "St. Marina", Varna, BGR; 2 Department of Clinical Laboratory, Medical University "Prof. Dr. Paraskev Stoyanov", Varna, BGR; 3 Clinic of Maxillofacial Surgery, University Hospital "St. Marina", Varna, BGR; 4 Department of General and Operative Surgery, Medical University "Prof. Dr. Paraskev Stoyanov", Varna, BGR; 5 Clinic of Nephrology and Dialysis, University Hospital "St. Marina", Varna, BGR; 6 Second Department of Internal Disease, Medical University "Prof. Dr. Paraskev Stoyanov", Varna, BGR

**Keywords:** abscess, c-reactive protein, head and neck, head and neck infection, maxillofacial surgery, mean platelet volume, neutrophil-to-lymphocyte ratio, non-odontogenic abscess, odontogenic abscess, procalcitonin

## Abstract

Introduction

Head and neck abscesses are categorized as either odontogenic, originating from dental infections, or non-odontogenic, arising from soft tissue inflammation. This article aimed to investigate inflammatory markers, including the neutrophil-to-lymphocyte ratio (NLR) and mean platelet volume (MPV), and compare them against established diagnostic parameters such as white blood cell (WBC) count, neutrophil count (Neu), lymphocyte count (Ly), platelet count (PLT), C-reactive protein (CRP), and procalcitonin (PCT).

Material and methods

Our retrospective analysis of a prospective study involved 80 patients, including 50 individuals (56% men) with an average age of 41.6±18.18 years diagnosed with odontogenic abscesses and 30 patients (66.7% men) with an average age of 44.53±15.49 years diagnosed with non-odontogenic head and neck abscesses, during the period from July 2021 to June 2022. The complete blood count provided data on WBC, Neu, Ly counts, MPV, and PLT using a 5-diff hematological analyzer. The MPV to PLT index (MPI) was calculated by dividing MPV by PLT, neutrophil-to-lymphocyte ratio (NLR) by dividing Neu by Ly, and platelet-to-white blood cell ratio (PWR) by dividing PLT to WBC. A latex-enhanced immunoturbidimetric assay was used to determine CRP (mg/L) on Cobas 6000 and PCT (ng/mL) on Advia 1800.

Results

PLT, PCT, and Ly were lower in patients with odontogenic abscesses compared to those with non-odontogenic abscesses (p<0.05), while CRP and Neu were significantly elevated in the odontogenic abscess group (p<0.05). In the non-odontogenic abscess group, men presented higher CRP levels (50.39±64.99 ng/mL versus 9.77±8.36 mg/L), MPI (0.036±0.017 ng/mL versus 0.029±0.01 ng/mL), and NLR (5.6±5.66 versus 3.09±2.2) compared to women (p<0.05). In contrast, women exhibited higher PLT values (390.78±197.129x10^9^/L versus 220.33±55.153x10^9^/L) and PWR (43.88±32.72 versus 23.86±9.34) compared to men (p<0.05). A significant combination of Ly% and Neu% was identified for predicting the MPV value in the non-odontogenic abscess group (R²=0.126, p=0.042), NLR and PWR as a predictor of CRP in both study groups (p<0.05) (R²=0.3 for the non-odontogenic abscess group, R²=0.448 for the odontogenic abscess group), and NLR and PWR as predictors for PCT values in those with non-odontogenic abscesses (R²=0.239, p=0.025).

Discussion

Despite the expected association between elevated MPV and heightened inflammatory responses, our findings revealed an inverse correlation between MPV and Neu and Ly in patients with non-odontogenic abscesses. This indicates complex interactions in immune responses during inflammation and underscores the need for further investigation to clarify MPV's role across different abscess types. Integrating traditional inflammatory markers such as CRP and PCT with indices such as NLR and MPV can enhance our understanding of inflammatory responses and improve predictions regarding patient outcomes in head and neck abscesses.

Conclusion

Our study confirms the value of combining traditional inflammatory markers such as CRP and PCT with indices such as NLR and MPV to assess the severity of infections in head and neck abscesses. These biomarkers provide significant insights into inflammatory responses and may improve patient outcome predictions. However, further research with larger, diverse populations is needed to fully integrate these markers into routine clinical practice for better management of such infections.

## Introduction

Abscesses of the head and neck are the most common emergency conditions leading to hospitalizations in the clinics of maxillofacial surgery [1]. If the purulent infection is not promptly treated and controlled, it can spread over a large area, a condition called phlegmon, lead to serious and life-threatening conditions such as mediastinitis, cavernous sinus thrombosis, and sepsis, and even be lethal [2]. Their etiology can be very different, but most authors conditionally divide them into two main groups: odontogenic and non-odontogenic [3]. Odontogenic abscesses occur as a result of inflammation of dental tissues and the periodontium, during which various pathogens enter the jawbone, first through its spongiosa and corticalis, and then through the periosteum of the jaw, they pass into the soft tissues of the head and neck [4]. Non-odontogenic abscesses of the maxillofacial region occur most often as a result of inflammation of the soft tissues in this area, suppurating skin cysts, impaired integrity of the skin or mucosal covering, or acute and exacerbated chronic infections of the upper respiratory tract [5]. Imbalances in the oral microbiome can lead to immune and systemic manifestations. This is usually related to the spread of microorganisms into the bloodstream or the lymph [6].

The significant number of individuals affected by these emergency conditions, coupled with their prolonged hospital stays, including in intensive care units, places a considerable financial strain on healthcare systems. This situation necessitates ongoing research and analysis, as well as the exploration of new and diverse inflammation markers.

One such biomarker is the neutrophil-to-lymphocyte ratio (NLR), which is easily accessible and serves as a sensitive indicator of inflammation and stress, although it lacks high specificity. NLR has shown promise in predicting outcomes across various diseases, including cardiovascular, cancer, and renal conditions [7]. However, research focusing on NLR in odontogenic infections is sparse. Elevated NLR levels often indicate abnormal inflammatory responses, as systemic inflammation can lead to lymphocyte redistribution and resulting lymphopenia [8]. This biomarker can be obtained from a complete blood count and signifies active infection. Consequently, a high NLR is likely to be present in patients with deep neck space infections [7].

Furthermore, C-reactive protein (CRP) and procalcitonin (PCT) levels are also elevated in patients exhibiting abnormal inflammatory responses [9]. The combination of these inflammatory markers may yield a more accurate assessment of infection severity in head and neck abscesses of both odontogenic and non-odontogenic origins.

Another key indicator, mean platelet volume (MPV), reflects platelet (PLT) function, production rate, and stimulation [10]. Routine hematological evaluations facilitate its measurement, making it an accessible tool for clinicians. Studies have demonstrated that MPV correlates significantly with disease progression, including septicemia [8,10]. Elevated MPV levels often indicate heightened PLT activation and are associated with severe inflammatory responses, emphasizing its role in managing critical health conditions.

This original article aims to fill the research gap by investigating previously unstudied inflammatory markers in odontogenic and non-odontogenic head and neck abscesses, specifically MPV and NLR. It will also compare and analyze these markers against well-established diagnostic parameters, including white blood cell (WBC) count, neutrophil count (Neu), lymphocyte count (Ly), PLT, CRP, and PCT. We hypothesize that a combination of hematological and inflammatory parameters derived from blood test data can effectively evaluate the severity of infection.

## Materials and methods

This report presents the findings of a prospective study conducted from July 1, 2021, to June 30, 2022. The study follows an observational, comparison group design and was approved by the Institutional Review Board of the Medical University "Prof. Dr. Paraskev Stoyanov" in Varna, Bulgaria. The study included 80 patients with head and neck abscesses who were hospitalized and urgently operated on at the Clinic of Maxillofacial Surgery at University Hospital "St. Marina," Varna, Bulgaria. In all cases, the diagnosis of abscess was confirmed through both clinical examination and surgical intervention, during which varying amounts of pus were evacuated. The patients were divided into two groups based on the etiology of their condition: 50 patients with odontogenic abscesses and 30 with non-odontogenic abscesses of the head and neck.

The inclusion criteria for participation in the study were evacuation of pus during the incision, age 18 years and older, and preserved complete medical documentation allowing the clear demarcation of odontogenic and non-odontogenic etiology of the disease.

The exclusion criteria included individuals under 18 years of age, those lacking preserved medical documentation, and patients with conditions or diseases that might artificially elevate levels of PCT, CRP, WBC count, and PLT count. Such conditions comprised parasitic infestations, recent significant trauma or surgical interventions, paraneoplastic syndromes, burns, oncological disorders, medications that enhance cytokine production, tissue hypoperfusion, cardiogenic shock, bronchial asthma, and pulmonary pneumonia. Furthermore, in terms of complete blood count parameters, the exclusion criteria extended to include immunosuppression, pregnancy, confirmed diagnoses of blood disorders (such as leukemia, thalassemia, immune thrombocytopenia, and multiple myeloma), and recent treatments involving anticoagulants, antiaggregants, granulocyte boosters, or clinical transfusion therapies.

Blood samples were collected preoperatively from all participants following the diagnosis of head and neck abscesses. Whole blood was obtained in vacutainers containing dipotassium ethylenediaminetetraacetic acid (K_2_EDTA) as an anticoagulant. Routine complete blood count analyses were performed using an automated 5-Diff hematology analyzer (ADVIA 2020, Siemens Healthineers, Erlangen, Germany) to derive total counts for WBC, neutrophils (Neu), lymphocytes (Ly), and platelets (PLT). This advanced flow cytometry-based system employs light scatter, differential leukocyte lysis, and myeloperoxidase and oxazine 750 staining to produce a comprehensive blood cell profile. The PLT count was determined using the impedance principle, and the mean platelet volume (MPV) was calculated automatically by dividing plateletcrit by the PLT count. The results were reported in the following units: WBC in 10^9^/L, PLT in 10^9^/L, and both Neu and Ly counts in either 10^9^/L or percentage (%). Additionally, the MPV to PLT index (MPI) was calculated by dividing MPV by PLT, expressed as a numerical value. The neutrophil-to-lymphocyte ratio (NLR) and platelet-to-white blood cell ratio (PWR) were also calculated by dividing the Neu count by the Ly count and PLT by WBC count, respectively, and expressed as numerical data.

For the analyses of C-reactive protein (CRP) and procalcitonin (PCT), serum was separated from blood collected in vacutainers equipped with gel separators by centrifugation at 2,500 G for 15 minutes. CRP levels were quantified using immunoturbidimetric analysis with latex-enhanced particles on the Cobas^® ^6000 platform (Roche Diagnostics Corporation, Indianapolis, IN). PCT levels were measured using a latex-enhanced immunoturbidimetric assay on the ADVIA 1800 biochemical analyzer (Siemens Healthineers), utilizing a reagent kit provided by Diazyme Laboratories, Inc. (Poway, CA). The results were reported in mg/L for CRP and ng/mL for PCT.

Statistical analyses were performed using the SPSS software package version 19 (IBM Corp., Armonk, NY) on a Windows 10.0 platform (Microsoft Corp., Redmond, WA). Numerical data were presented as mean values ± standard deviation (SD). Initially, descriptive statistics were utilized to ascertain the central tendency and dispersion of the data. Pearson's correlation coefficient (r) was applied to conduct correlation analyses, assessing the linear relationships between variables. Both univariate and multivariate linear regression analyses were carried out to explore the connections between independent and dependent variables. Additionally, factor analysis, along with independent samples T-tests, was performed to identify significant differences in sample means. Nonparametric statistical methods, including the Chi-square test of independence, were used for the analysis of nominal data. A significance level (α) of 0.05 was upheld throughout all analyses, rejecting the null hypothesis for p-values below α (p<0.05).

## Results

Gender distribution was similar across both groups (χ²=0.889, p=0.346): 56% men and 44.4% women with odontogenic abscesses and 66.7% men and 33.3% women with non-odontogenic abscesses of the head and neck. The average age in the odontogenic abscess group was 41.6±18.18 years, while in the non-odontogenic abscess group, it was 44.53±15.49 years, with no significant difference observed between them (F=1.345, p=0.25).

Table 1 provides a summary of the patient data. PLT, PCT, and Ly were lower in patients with odontogenic abscesses compared to those with non-odontogenic abscesses (p<0.05). On the other hand, CRP and Neu were significantly elevated in patients with odontogenic abscesses (p<0.05) as shown in Table 1.

**Table 1:** Mean values of laboratory parameters in clinical groups with head and neck abscesses An independent samples T-test was performed to determine significant differences between sample means. P-values below 0.05 are considered statistically significant, while those below 0.09 indicate significance at a 90% confidence level. The results for MPI, PWR, and NLR are presented as numerical data. n: number, SD: standard deviation, WBC: white blood cells, L: liter, Neu: neutrophil count, Ly: lymphocyte count, MPV: mean platelet volume, fL: femtoliter, PLT: platelet count, MPI: mean platelet volume-to-platelet count ratio, CRP: C-reactive protein, mg: milligram, mL: milliliter, PCT: procalcitonin, ng: nanogram, NLR: neutrophil count to lymphocyte count, PWR: platelet count to leukocyte count

Studied marker	Patients with odontogenic abscesses (n=50)		Patients with non-odontogenic abscesses (n=30)		Reference range	F/T-test	P-value
	Mean value	SD	Mean value	SD			
WBC (10^9^/L)	11.17	4.429	10.33	3.19	3.79-10	1.457	0.231
Neu (10^9^/L)	8.45	4.34	7.24	3.23	1.78-7	1.523	0.221
Neu (%)	73.672	11.49	68.11	12.45	39-77	2.029	0.046
Ly (%)	16.59	9.27	22.37	10.57	20-44	-2.557	0.013
Ly (10^9^/L)	1.64	0.7	2.13	0.91	1.07-3.12	-2.712	0.008
MPV (fL)	8.63	1.21	8.57	1.36	6.0-10	0.083	0.774
PLT (10^9^/L)	268.38	80.46	301.77	133.02	140-440	4.619	0.035
MPI (MPV/PLT)	0.038	0.028	0.034	0.015	-	0.015	0.902
CRP (mg/L)	104.94	111.75	36.85	56.29	0-5	9.564	0.003
PCT (ng/mL)	0.816	1.02	1.26	1.53	0-0.05	7.994	0.006
NLR (Neu/Ly)	6.68	5.73	4.7	4.83	-	1.686	0.198
PWR (PLT/WBC)	27.44	12.5	37.37	22.89	-	0.633	0.429

No significant gender differences were identified in the mean levels of all analyzed parameters within the odontogenic abscess group (p>0.05). However, among patients with non-odontogenic abscesses, men presented significantly higher mean levels of CRP, MPI, and NLR compared to women (p<0.05). In contrast, women exhibited significantly higher mean values of PLT and PWR compared to men (p<0.05). No significant gender-based differences were found for the remaining variables within the non-odontogenic abscess group (p>0.05) as shown in Table 2.

**Table 2:** Mean values of laboratory parameters for men and women in clinical groups with head and neck abscesses An independent samples T-test was performed to determine significant differences between sample means. P-values below 0.05 were considered statistically significant, while those below 0.09 indicated significance at a 90% confidence level. The results for MPI, PWR, and NLR were presented as numerical data. n: number, SD: standard deviation, WBC: white blood cells, L: liter, Neu: neutrophil count, Ly: lymphocyte count, MPV: mean platelet volume, fL: femtoliter, PLT: platelet count, MPI: mean platelet volume-to-platelet count ratio, CRP: C-reactive protein, mg: milligram, PCT: procalcitonin, ng: nanogram, NLR: neutrophil count to lymphocyte count, PWR: platelet count to leukocyte count

Studied group	Studied marker	Gender	Mean value ± SD	F/T-test	P-value
Patients with odontogenic abscesses (n=50)	WBC (10^9^/L)	Male versus female	11.69±5.05 versus 10.49±3.48	1.603	0.212
	Neu (10^9^/L)	Male versus female	8.87±4.95 versus 7.9±3.44	1.32	0.256
	Neu (%)	Male versus female	74.27±11.55 versus 72.91±11.66	0.066	0.685
	Ly (10^9^/L)	Male versus female	1.62±0.69 versus 1.65±0.73	0.086	0.77
	Ly (%)	Male versus female	16.09±9.16 versus 17.24±9.63	0.725	0399
	MPV (fL)	Male versus female	8.68±1.15 versus 8.58±1.3	0.005	0.944
	PLT (10^9^/L)	Male versus female	262.18±79.77 versus 276.27±82.51	0.215	0.645
	MPI (MPV/PLT)	Male versus female	0.041±0.036 versus 0.034±0.011	1.754	0.192
	PWR (PLT/WBC)	Male versus female	26.09±12.5 versus 29.35±13.23	0.007	0.932
	NLR (Neu/Ly)	Male versus female	6.97±5.29 versus 6.81±6.323	0.036	0.849
	PCT (ng/mL)	Male versus female	0.937±1.165 versus 0.661±0.799	2.496	0.121
	CRP (mg/L)	Male versus female	112.73±127.61 versus 95.034±89.56	2.075	0.156
Patients with non-odontogenic abscesses (n=30)	WBC (10^9^/L)	Male versus female	10.446±3.053 versus 10.093±3.614	0.13	0.721
	Neu (10^9^/L)	Male versus female	7.55±3.17 versus 6.633±3.43	0.066	0.799
	Neu (%)	Male versus female	69.99±12.33 versus 64.36±12.43	0.069	0.794
	Ly (10^9^/L)	Male versus female	1.925±0.831 versus 2.535±0.966	1.357	0.254
	Ly (%)	Male versus female	20.185±10.19 versus 26.74±10.44	0.156	0.696
	MPV (fL)	Male versus female	8.665±1.544 versus 8.37±0.929	1.504	0.23
	PLT (10^9^/L)	Male versus female	299.8±156.26 versus 305.7±73.74	3.99	0.05
	MPI (MPV/PLT)	Male versus female	0.0361±0.0171 versus 0.0293±0.0102	4.38	0.046
	PWR (PLT/WBC)	Male versus female	28.92±11.423 versus 32.64±10.788	0.294	0. 592
	NLR (Neu/Ly)	Male versus female	5.559±5.364 versus 3.089±2.203	3.294	0.05
	PCT (ng/mL)	Male versus female	1.131±1.695 versus 1.526±1.179	0.151	0.701
	CRP (mg/L)	Male versus female	50.396±64.99 versus 9.77±8.36	11.642	0.002

Significant correlations were observed between WBC and Neu, NLR, and CRP levels in both study groups, indicating a direct and strong relationship (all with p<0.005) as shown in Table 3 and Table 4. Additionally, a significant negative correlation was found between WBC and Ly% in both groups, with a strong relationship (p<0.005). We did not find a significant correlation between WBC and the absolute count of Ly in either study group (p>0.05) as shown in Table 3 and Table 4. Neu also showed strong correlations with Ly and CRP levels in the non-odontogenic abscess group and odontogenic abscess group (both with p<0.001). Ly correlated negatively with CRP levels in both study groups (p<0.05) (Table 3 and Table 4).

**Table 3:** Correlations between the analyzed laboratory parameters in the group of individuals with odontogenic abscesses of the head and neck Pearson correlation test was performed to determine significant differences between sample means. P-values below 0.05 were considered statistically significant, while those below 0.09 indicated significance at a 90% confidence level. The results for MPI, PWR, and NLR were presented as numerical data. n: number, SD: standard deviation, WBC: white blood cells, L: liter, Neu: neutrophil count, Ly: lymphocyte count, MPV: mean platelet volume, fL: femtoliter, PLT: platelet count, MPI: mean platelet volume-to-platelet count ratio, CRP: C-reactive protein, mg: milligram, PCT: procalcitonin, ng: nanogram, mL: milliliter, NLR: neutrophil count to lymphocyte count, PWR: platelet count to leukocyte count

Studied marker		WBC (10^9^/L)	Neu (10^9^/L)	Neu (%)	Ly (10^9^/L)	Ly (%)	MPV (fL)	PLT (10^9^/L)	MPI	PWR	NLR	PCT (ng/mL)	CRP (mg/L)
Neu (10^9^/L)	Pearson correlation, p-value	0.976, 0.000	/										
Neu (%)	Pearson correlation, p-value	0.598, 0.000	0.728, 0.000	/									
Ly (10^9^/L)	Pearson correlation, p-value	-0.031, 0.83	-0.205, 0.154	-0.598, 0.000	/								
Ly (%)	Pearson correlation, p-value	-0.541, 0.000	-0.659, 0.000	-0.871, 0.000	0.777, 0.000	/							
MPV (fL)	Pearson correlation, p-value	-0.078, 0.588	-0.135, 0.349	-0.33, 0.019	0.221, 0.123	0.352, 0.012	/						
PLT (10^9^/L)	Pearson correlation, p-value	-0.026, 0.856	-0.045, 0.754	-0.074, 0.611	0.029, 0.841	0.046, 0.752	0.028, 0.848	/					
MPI	Pearson correlation, p-value	-0.054, 0.7	-0.092, 0.523	-0.219, 0.127	0.243, 0.089	0.25, 0.08	0.299, 0.035	-0.65, 0.000	/				
PWR	Pearson correlation, p-value	-0.702, 0.000	-0.705, 0.000	-0.58, 0.000	-0.017, 0.909	0.435, 0.002	0.15, 0.297	0.624, 0.000	-0.399, 0.004	/			
NLR	Pearson correlation, p-value	0.424, 0.003	0.515, 0.000	0.509, 0.000	-0.722, 0.000	-0.804, 0.000	-0.191, 0.185	-0.024, 0.185	-0.123, 0.397	-0.19, 0.187	/		
PCT (ng/mL)	Pearson correlation, p-value	0.021, 0.893	0.056, 0.698	0.154, 0.286	-0.047, 0746	-0.07, 0.628	0.074, 0.608	0.145, 0.315	-0.044, 0.761	0.025, 0.862	0.058, 0.691	/	
CRP (mg/L)	Pearson correlation, p-value	0.565, 0.000	0.587, 0.000	0.445, 0.001	-0.252, 0.078	-0.459, 0.001	-0.45, 0.813	0.223, 0.237	-0.068, 0.722	-0.434, 0.002	0.41, 0.003	0.204, 0.156	/

**Table 4:** Correlations between the analyzed laboratory parameters in the group of individuals with non-odontogenic abscesses of the head and neck Pearson correlation test was performed to determine significant differences between sample means. P-values below 0.05 were considered statistically significant, while those below 0.09 indicated significance at a 90% confidence level. The results for MPI, PWR, and NLR were presented as numerical data. n: number, SD: standard deviation, WBC: white blood cells, L: liter, Neu: neutrophil count, Ly: lymphocyte count, MPV: mean platelet volume, fL: femtoliter, PLT: platelet count, MPI: mean platelet volume-to-platelet count ratio, CRP: C-reactive protein, mg: milligram, PCT: procalcitonin, ng: nanogram, mL: milliliter, NLR: neutrophil count to lymphocyte count, PWR: platelet count to leukocyte count

Studied marker		WBC (10^9^/L)	Neu (10^9^/L)	Neu (%)	Ly (10^9^/L)	Ly (%)	MPV (fL)	PLT (10^9^/L)	MPI	PWR	NLR	PCT (ng/mL)	CRP (mg/L)
Neu (10^9^/L)	Pearson correlation, p-value	0.941, 0.000	/										
Neu (%)	Pearson correlation, p-value	0.567, 0.000	0.794, 0.000	/									
Ly (10^9^/L)	Pearson correlation, p-value	-0.416, 0.022	-0.324, 0.081	-0.769, 0.000	/								
Ly (%)	Pearson correlation, p-value	-0.559, 0.001	-0.78, 0.000	-0.988, 0.000	0.774, 0.000	/							
MPV (fL)	Pearson correlation, p-value	-0.216, 0.253	-0.213, 0.258	-0.276, 0.14	0.06, 0.754	0.262, 0.161	/						
PLT (10^9^/L)	Pearson correlation, p-value	0.475, 0.008	0.41, 0.024	0.189, 0.316	0.127, 0.504	-0.172, 0.364	-0.48, 0.007	/					
MPI	Pearson correlation, p-value	-0.385, 0.035	-0.293, 0.116	-0.102, 0.594	0.074, 0.697	-0.213, 0.259	0.781, 0.000	-0.835, 0.000	/				
PWR	Pearson correlation, p-value	-0.284, 0.129	-0.293, 0.116	-0.298, 0.205	0.099, 0.601	0.264, 0.159	-0.343, 0.063	0.663, 0.000	-0.616, 0.004	/			
NLR	Pearson correlation, p-value	0.523, 0.003	0.73, 0.000	0.809, 0.000	-0.691, 0.000	-0.795, 0.000	-0.099, 0.601	0.127, 0.505	-0.074, 0.805	-0.18, 0.187	/		
PCT (ng/mL)	Pearson correlation, p-value	0.294, 0.115	0.324, 0.081	0.255, 0.173	-0.128, 0.5	-0.246, 0.189	-0.87, 0.648	-0.118, 0.535	-0.023, 0.906	-0.268, 0.152		/	
CRP (mg/L)	Pearson correlation, p-value	0.504, 0.004	0.608, 0.000	0.583, 0.000	-0.416, 0.022	-0.592, 0.001	-0.045, 0.813	0.223, 0.237	-0.068, 0.722	-0.102, 0.593	0.665, 0.000	0.301, 0.106	/

In the non-odontogenic abscess group, MPV correlated negatively with the relative count of Neu (r=-0.330, p=0.019) and positively with the relative count of Ly (r=0.352, p=0.012), although these correlations did not reach statistical significance in the odontogenic abscess group (Table 3 and Table 4). A significant negative correlation was found between MPV and PLT in the odontogenic abscess group, while PLT positively correlated with WBC in the same group (p<0.05). A strong positive correlation between CRP and NLR was noted in both study groups (p<0.05). Additionally, a strong negative correlation between CRP and PWR was observed in the non-odontogenic abscess group (r=-0.434, p=0.002). A significant positive correlation between PCT and NLR was also found in the same group (r=0.464, p=0.01). The association between PCT and PWR in the non-odontogenic abscess group was notable but did not reach statistical significance (r=-0.268, p=0.152). Finally, a notable association was found between CRP and PCT in both groups, although not reaching statistical significance (non-odontogenic abscess group: r=0.204, p=0.155; odontogenic abscess group: r=0.301, p=0.106) (Table 3 and Table 4).

To establish the linear combination between MPV and the analyzed variables (Ly% and Neu%), multiple linear regression analysis was applied. A statistically significant combination of the variables Ly% and Neu% was identified for predicting the value of MPV in individuals with non-odontogenic abscesses (F=3.4, p=0.042). The constructed model was statistically insignificant for the odontogenic abscess group (F=1.193, p=0.319). In the non-odontogenic abscess group, the regression constant α=8.793 (p=0.002). The equation found for the relationship between the variables was MPV (fL)=8.793+0.035×Ly(%)-0.01×Neu(%).

**Figure 1:**
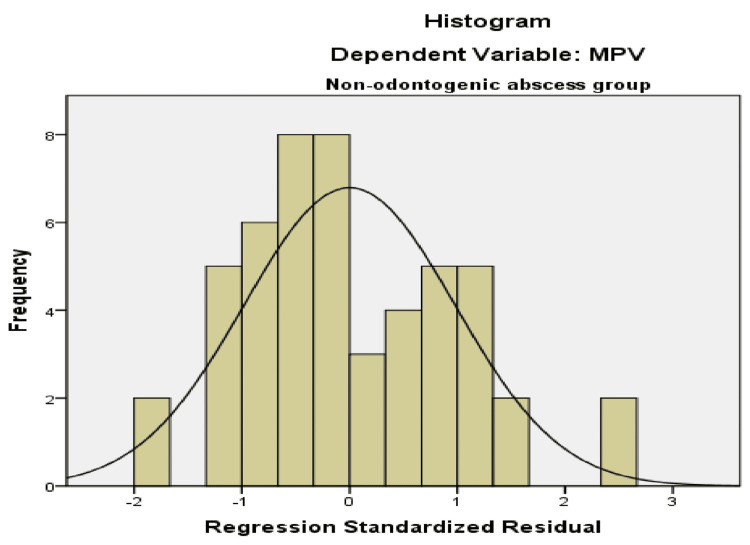
Histogram for normal distribution as a prerequisite for regression analysis in individuals with non-odontogenic abscess Dependent variable: MPV Predictors: Neu% and Ly% MPV: mean platelet volume, Neu: neutrophils, Ly: lymphocytes

**Figure 2:**
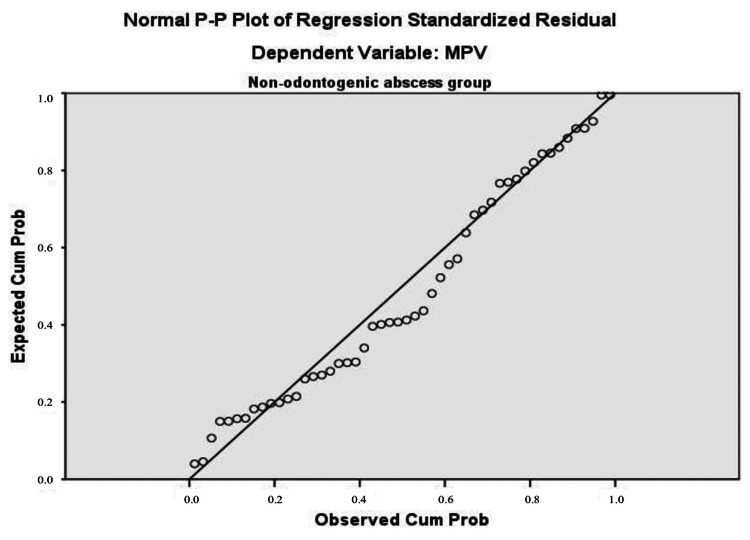
P-P plot for normal distribution as a prerequisite for regression analysis in individuals with non-odontogenic abscess Dependent variable: MPV Predictors: Neu% and Ly% MPV: mean platelet volume, Neu: neutrophils, Ly: lymphocytes

The strength of the relationship between the combination of independent variables (Ly and Neu) and the dependent variable (MPV) was moderate (R=0.356). The adjusted coefficient of determination (adjusted R²) was R²=0.126, suggesting that 12.6% of the variation in MPV values can be explained by the presented regression model. To verify the key assumption of homogeneity of variance in multiple regression analysis, standardized residual plots were presented. Statistical testing for normal distribution, along with a visual assessment of the histogram, confirmed a normal Gaussian distribution of MPV results in patients with non-odontogenic abscesses (Figure 1). The P-P plot shows that the "thick" line lies very close to the "thin" diagonal (Figure 2).

Furthermore, a statistically significant combination of the variables NLR and PWR was identified for predicting the value of CRP in individuals with non-odontogenic abscess (F=10.062, p<0.001) and in the odontogenic abscess group (F=10.961, p<0.001). The equations found for the relationship between the variables were CRP (mg/L)=147.763+6.658×PWR-3.225×NLR for patients with non-odontogenic abscess and CRP (mg/L)=0.399×PWR+8.278×NLR-3.225 for patients with odontogenic abscess.

The strength of the relationship between the combination of independent variables (PWR and NLR) and the dependent variable (CRP) was moderate (non-odontogenic abscess group: R=0.548, odontogenic abscess group: R=0.669). The adjusted coefficients of determination (adjusted R²) were R²=0.30 and R2=0.448, suggesting that 30% of the variation in CRP values can be explained by the presented regression model in patients with non-odontogenic abscess and 44.8% in patients with odontogenic abscess, respectively.

**Figure 3:**
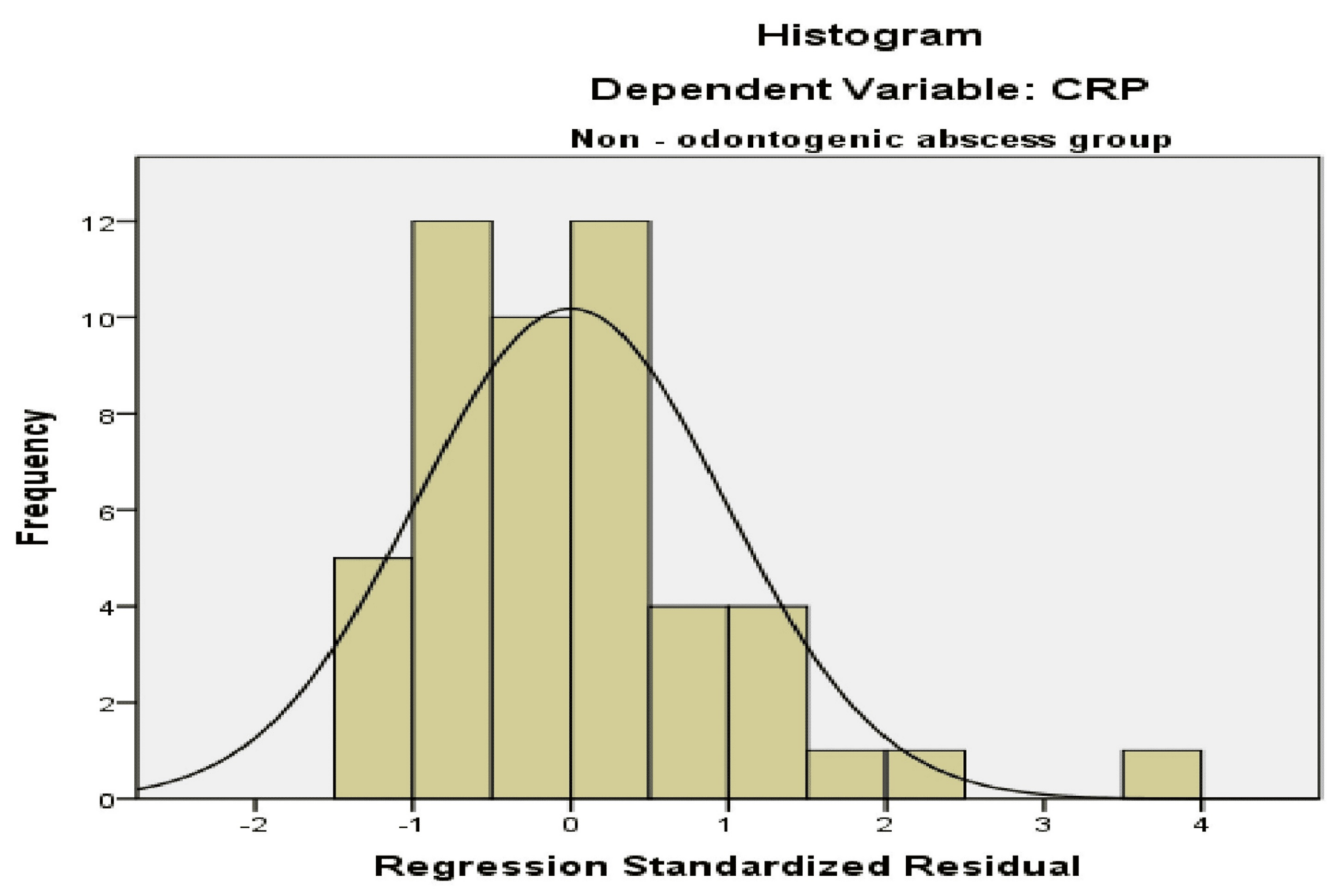
Histogram for normal distribution as a prerequisite for regression analysis in the non-odontogenic abscess group Dependent variable: CRP Predictors: PWR and NLR CRP: C-reactive protein, PWR: platelet count to leukocyte count, NLR: neutrophil count to lymphocyte count

**Figure 4:**
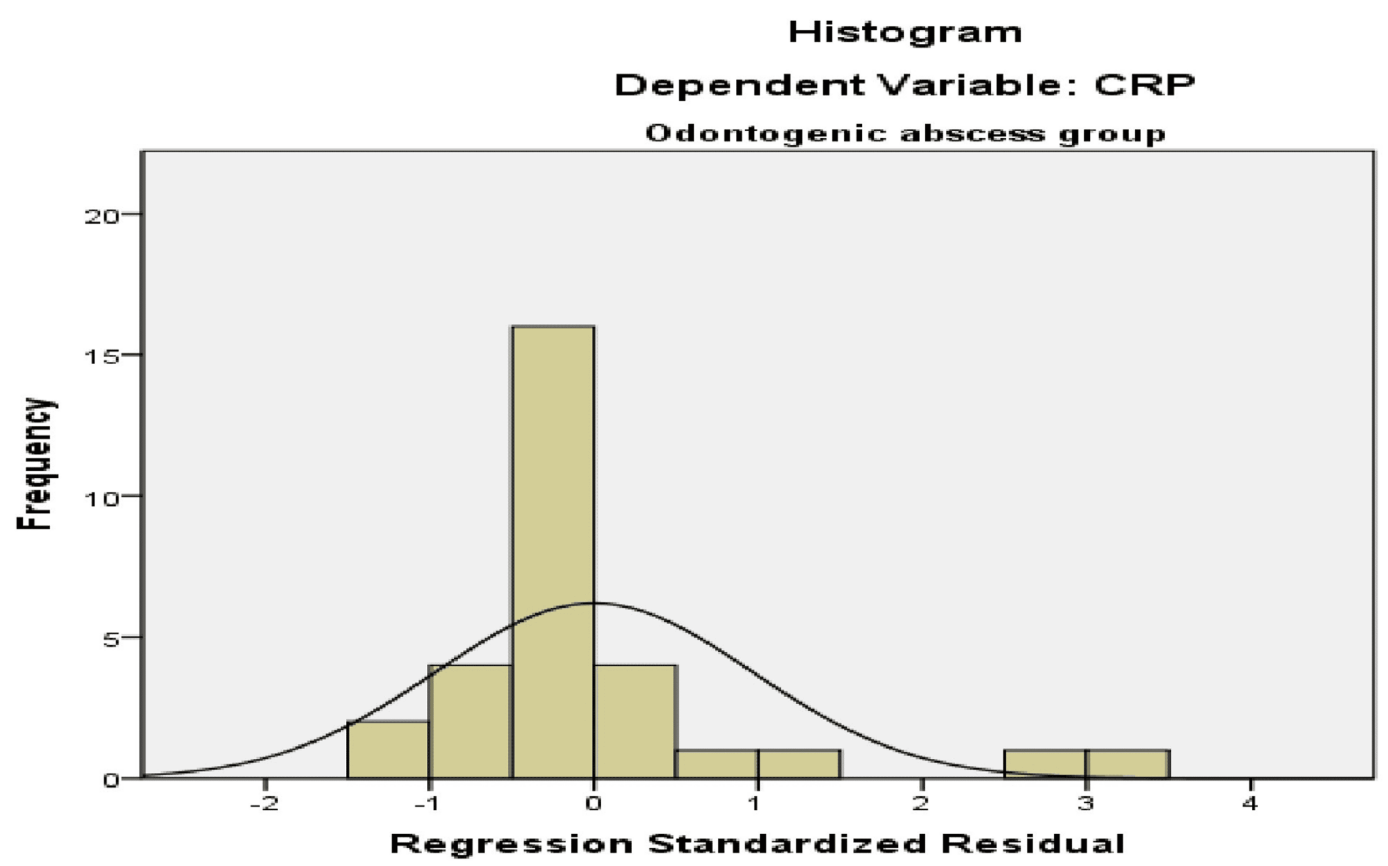
Histogram for normal distribution as a prerequisite for regression analysis in the odontogenic abscess group Dependent variable: CRP Predictors: PWR and NLR CRP: C-reactive protein, PWR: platelet count to leukocyte count, NLR: neutrophil count to lymphocyte count

**Figure 5:**
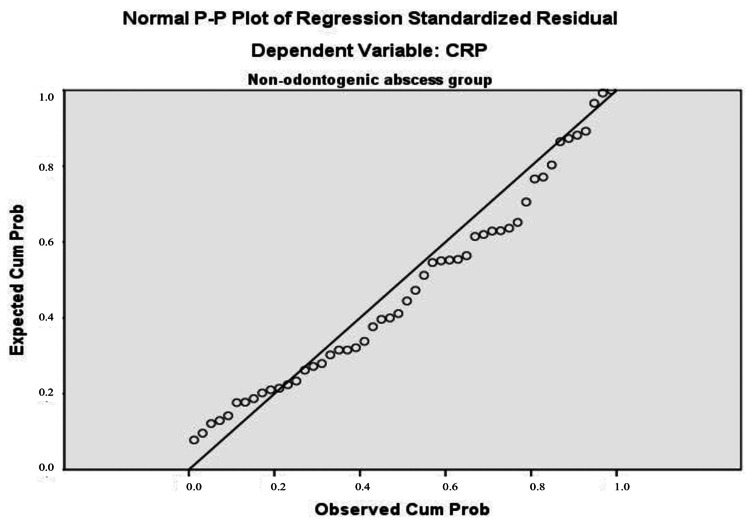
P-P plot for normal distribution as a prerequisite for regression analysis in the non-odontogenic abscess group Dependent variable: CRP Predictors: PWR and NLR CRP: C-reactive protein, PWR: platelet count to leukocyte count, NLR: neutrophil count to lymphocyte count

**Figure 6:**
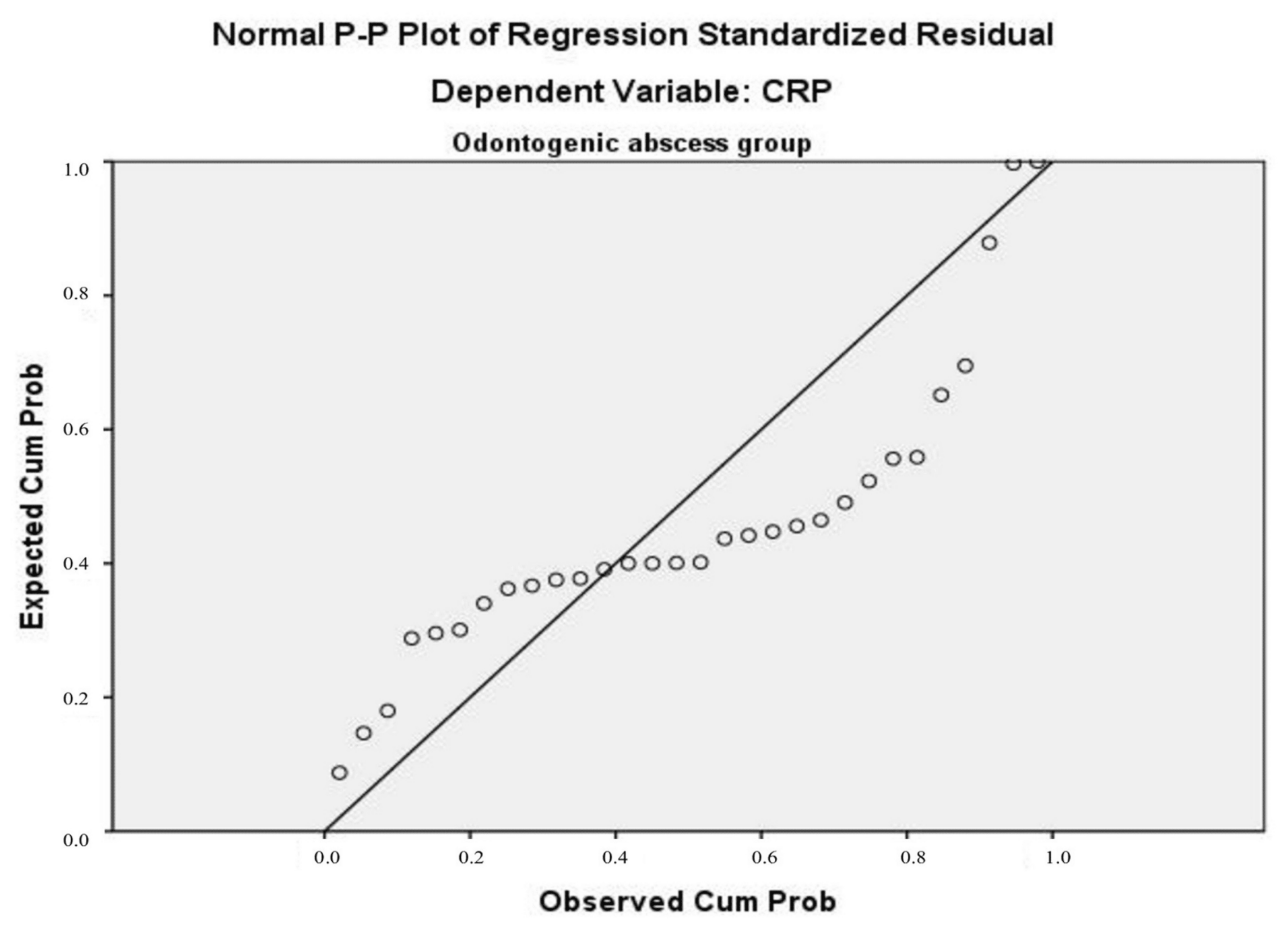
P-P plot for normal distribution as a prerequisite for regression analysis in the odontogenic abscess group Dependent variable: CRP Predictors: PWR and NLR CRP: C-reactive protein, PWR: platelet count to leukocyte count, NLR: neutrophil count to lymphocyte count

To verify the key assumption of homogeneity of variance in multiple regression analysis, standardized residual plots were presented. Statistical testing for normal distribution, along with a visual assessment of the histogram, confirmed a normal Gaussian distribution of CRP results (Figure 3 and Figure 4). The P-P plot shows that the "thick" line lies very close to the "thin" diagonal (Figure 5 and Figure 6).

A statistically significant combination of the variables NLR and PWR was also identified for predicting the value of PCT only in individuals with non-odontogenic abscess (F=4.23, p=0.025) with an adjusted R²=0.239, suggesting that 23.9% of the variation in PCT values can be explained by the presented regression model. The equation found for the relationship between the variables was PCT(ng/mL)=1.252-0.021×PWR+0.139×NLR.

**Figure 7:**
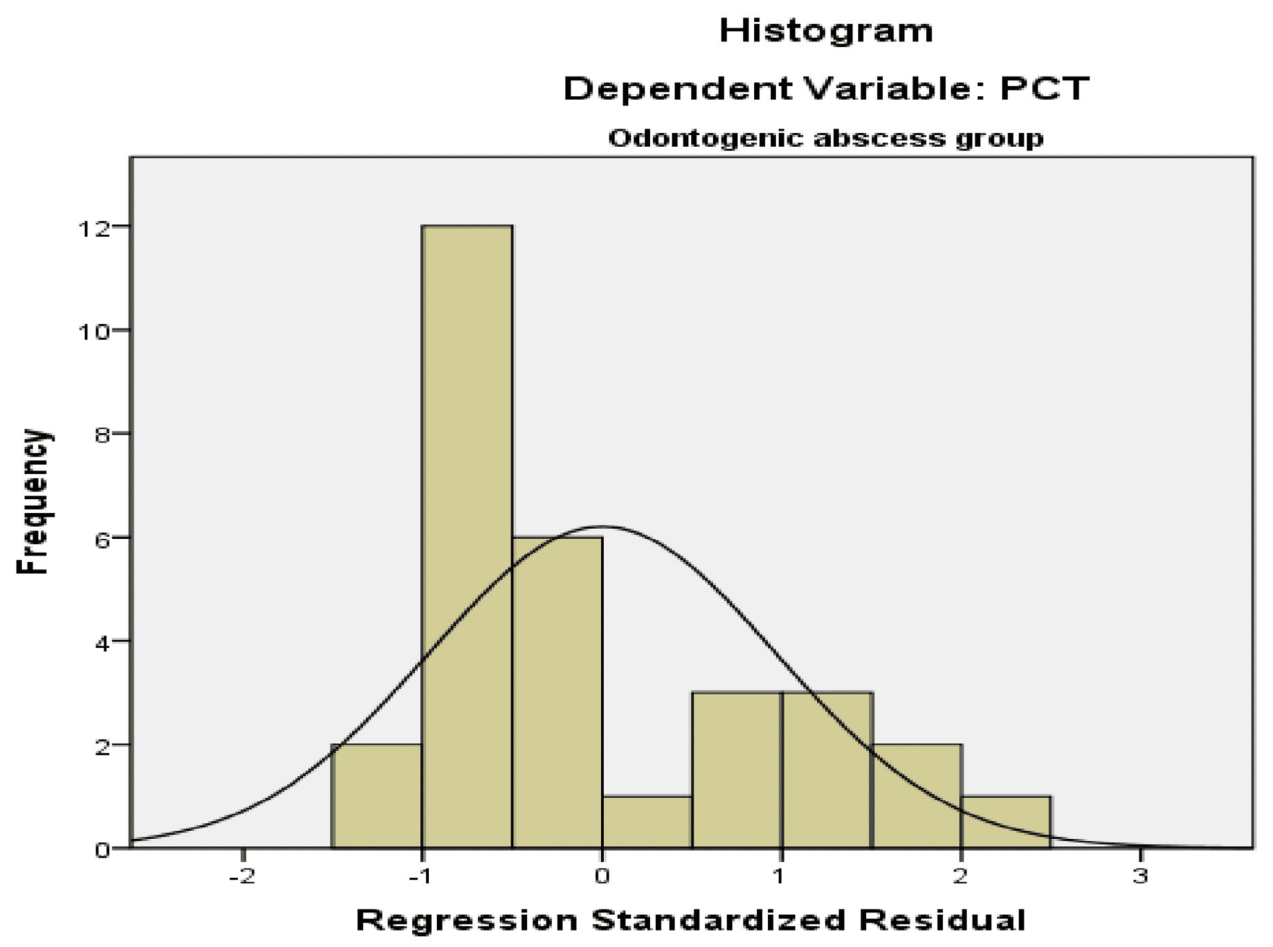
Histogram for normal distribution as a prerequisite for regression analysis Dependent variable: PCT Predictors: NLR and PWR PCT: procalcitonin, NLR: neutrophil count to lymphocyte count, PWR: platelet count to leukocyte count

**Figure 8:**
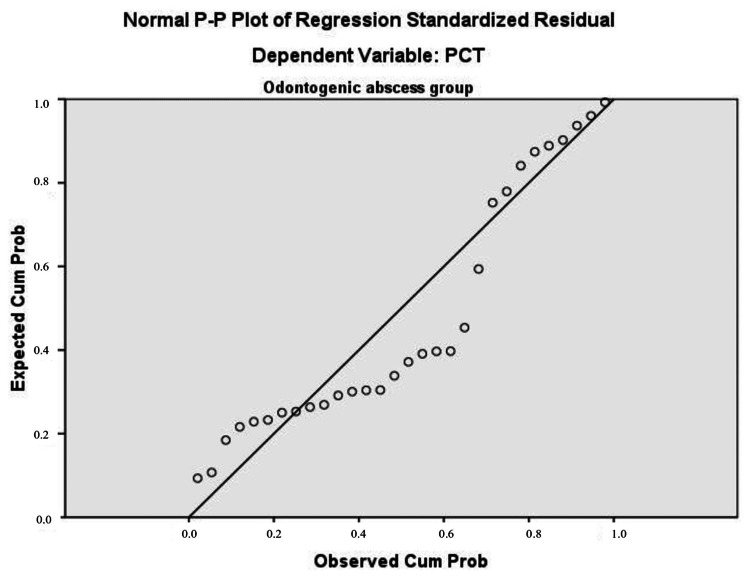
P-P plot for normal distribution as a prerequisite for regression analysis Dependent variable: PCT Predictors: NLR and PWR PCT: procalcitonin, NLR: neutrophil count to lymphocyte count, PWR: platelet count to leukocyte count

The histogram and P-P plot for normal distribution as a prerequisite for regression analysis are presented in Figure 7 and Figure 8, respectively.

## Discussion

In line with findings from other studies, our research indicates that odontogenic and non-odontogenic infections are most commonly observed in individuals aged approximately 40±20 years, with a slight male predisposition [7-10]. These results demonstrate that similar risk factors exist for both odontogenic and non-odontogenic infections.

In our study, patients with both odontogenic and non-odontogenic infections demonstrated elevated levels of CRP, PCT, WBC, and Neu count, consistent with findings from previous research [8,9,11,12]. CRP is the most commonly used inflammatory marker in assessing the severity of diseases, including odontogenic and non-odontogenic head and neck infections, as it responds quickly to inflammation and can detect changes in the patient's condition [9].

In comparison to earlier studies that reported similar CRP levels, we observed significantly higher values in our study, as well as a higher relative Neu count in the group with odontogenic abscesses compared to the group with non-odontogenic abscesses [9]. Specifically, the average CRP value in the group with odontogenic abscesses was 104.94±111.75 mg/L, which is almost three times higher than the value observed in the non-odontogenic abscess group (36.85±56.29 mg/L) (p=0.003). Additionally, Neu and CRP levels were typically elevated, while Ly counts were decreased in patients who developed abnormal inflammatory responses, a finding that we also confirmed in our study. According to Rosca et al. (2023), the association between CRP and NLR increases the risk of severe odontogenic infections by 7.28 times [13]. A receiver operating characteristic (ROC) analysis of the CRP-NLR showed an area under the curve (AUC) of 0.889, with high sensitivity (79.6%) and specificity (85.1%) for predicting severe odontogenic infections, based on biomarkers measured at the time of hospital admission (p<0.001) [13]. Kamiński et al. (2024) noted that these findings are associated with the higher morbidity observed in patients with odontogenic infections compared to those with non-odontogenic infections [9].

PCT begins to increase in concentration two to four hours after bacterial toxins start acting, reaching its peak plasma levels 8-24 hours later [11,12]. In contrast, CRP starts elevating its plasma concentration five to six hours after the etiological factor begins, reaching its highest plasma levels 35-50 hours afterward. Additionally, the plasma half-life of PCT is 24 hours, while that of CRP is 19 hours, meaning PCT normalizes in the blood more quickly after the infection is resolved [11,12]. This quicker response time of PCT suggests that it may be more precise than CRP in determining the progression of purulent inflammations and serve as a better indicator for their prognosis.

Numerous studies comparing PCT and CRP in inflammatory diseases and their complications, such as sepsis, chronic obstructive pulmonary disease (COPD), bacterial meningitis, and febrile neutropenia in children, have reached the same conclusion [12,14-16]. Both our research and previous studies, despite focusing on different anatomical locations of inflammation, found a positive correlation between PCT and the established inflammation marker, CRP. However, in our study, the correlation between the acute-phase proteins did not reach statistical significance.

Interestingly, we observed higher PCT values in patients with non-odontogenic abscesses compared to those with odontogenic abscesses. This finding was unexpected. A potential explanation could lie in the heterogeneity of the studied subgroups (with odontogenic and non-odontogenic abscesses) and the differing clinical severity and spread of purulent exudate in the anatomical regions of the head and neck. Expanding the current study and including more patients will likely yield clearer results and enhance the practical application of these findings.

In line with previous studies, we did not observe significant differences in the examined parameters between male and female patients in the group with odontogenic abscesses [8,9]. However, in the group with non-odontogenic abscesses, we found significantly higher average values for CRP, MPI, and NLR in male patients compared to female patients. We did not find an explanation for this finding in the literature.

In addition, women with non-odontogenic abscesses showed significantly higher values of PLT and PWR compared to men in the same group. Gender dimorphism in platelet reactivity has been observed for nearly 50 years [17-19]. It is well established that, across all ages, women's platelets respond more strongly to stimulation by agonists compared to those of men, and women generally have a higher platelet count. The influence of sex hormones (estrogens, progesterone, or androgens) on PLT, or their indirect effects on the vascular system, may be the primary biological factors behind these differences [17].

Biino et al. hypothesized that puberty leads to a difference in PLT count [17]. On the other hand, Sabetta et al. emphasize that platelet reactivity changes during the menstrual cycle and after menopause [18]. Based on the average age of our patients, we expect the observed differences in PLT count to align with these hormonal influences. PLT expresses estrogen receptor β (ERβ) and androgen receptor (AR). However, reports on the effects of estrogens and testosterone on PLT have been contradictory. In general, 17β-estradiol (the most biologically active estrogen) is believed to exert a protective effect on vascular diseases, which is lost after menopause [19]. Consistently, at least two studies have demonstrated that in vitro administration of estradiol reduces PLT activation. However, research on the differences in platelet reactivity before and after menopause, and the in vivo use of hormone replacement therapy post-menopause, remains highly contradictory [18,19].

In our study, a statistically significant combination of the variables Ly% and Neu% was identified for predicting the MPV value in individuals with non-odontogenic abscesses, amounting to 12.6%. In the group of patients with odontogenic abscesses, the described relationship between Ly% and Neu% for predicting the value of MPV did not reach statistical significance. According to numerous authors, increased MPV is associated with neutrophilia and lymphocytopenia, indicating an intensified inflammatory response in the body [8,10,20]. This correlation highlights the role of PLT as active participants in the immune response during infections, where larger and more reactive PLT are produced, accompanied by an increase in the number of Neu and a decrease in Ly [20]. The reported mean values for MPV did not show significantly higher levels compared to the reference values in both clinical groups. Surprisingly, we found an inverse correlation between MPV and both Neu and Ly in individuals with non-odontogenic abscesses. Possible explanations for this phenomenon include the fact that during inflammatory or immune responses, both PLT and Ly can be activated. An elevated MPV typically indicates increased PLT activation and production, while elevated Ly levels reflect their role in the inflammatory process. The body produces larger PLT in response to infection, and Ly, particularly T-cells, are mobilized to combat the pathogen [8]. Conversely, in conditions such as autoimmune diseases or chronic infections, both elevated MPV and increased Ly may be observed. The persistent inflammatory environment may stimulate the production of larger, more reactive PLT, along with the proliferation of Ly [10].

In recent years, numerous inflammatory indices have been developed, including the MPV-to-PLT ratio (MPI), platelet-to-white blood cell ratio (PWR), and neutrophil-to-lymphocyte ratio (NLR). These indices encompass various components of the immune response, such as Neu, Ly, and PLT, and are often associated with improved prognosis and the prediction of patient outcomes [20]. By providing a comprehensive view of different aspects of the body's immune response to inflammation, these parameters enable more accurate assessments of disease severity. Among these, NLR is a well-established prognostic indicator that correlates independently with mortality in both the general population (hazard ratio (HR): 1.14, 95% confidence interval (CI): 1.10-1.17 per quartile of NLR) and specific disease groups, including sepsis, pneumonia, COVID-19, and cancer [20,21]. Numerous studies have explored the prognostic value of NLR, particularly in odontogenic infections. In 2024, Ghasemi et al. concluded in their systematic review of nine studies that an elevated NLR significantly correlates with the severity of odontogenic infections, length of hospital stay, and risk of complications [22]. Furthermore, Rosca et al. conducted a study involving 108 patients and determined that the combination of CRP and NLR serves as a reliable and accessible biomarker for predicting the severity of odontogenic infections, surpassing the predictive value of either indicator alone [13]. In our study, we found that 30% of the variation in CRP values can be explained by changes in NLR and PWR in patients with non-odontogenic abscesses, while this figure increases to 44.8% in patients with odontogenic abscesses. Additionally, we identified a statistically significant combination of NLR and PWR for predicting the value of PCT, which explains 23.9% of the variation in this acute phase indicator specifically in individuals with non-odontogenic abscesses. In the group of patients with odontogenic abscesses, the described relationship between NLR and PWR for predicting the value of PCT did not reach statistical significance. A possible explanation may stem again from the variability within the studied subgroups (odontogenic and non-odontogenic abscesses), as well as the differing clinical severity and distribution of purulent exudate across various anatomical regions of the head and neck.

Limitations

As limitations of the present study, we note that it was conducted only for a period of one year, that it included only patients aged 18 years and older, that it analyzed patients from only one clinic of one hospital, and that the two studied groups did not include a very large number of participants. This necessitates conducting additional clinical studies to verify the validity of the results achieved in order to develop strategies for their future application in the diagnosis, treatment, and follow-up of the outcome of the disease in patients with odontogenic and non-odontogenic abscesses of the head and neck.

## Conclusions

In conclusion, our study underscores the significance of evaluating various inflammatory markers to better understand and manage head and neck abscesses, both odontogenic and non-odontogenic. The findings confirm that traditional inflammatory markers, such as CRP and PCT, alongside indices such as NLR and MPV, are valuable in assessing the severity of infections. Elevated levels of these laboratory parameters provide crucial insights into the body's inflammatory response and can serve as reliable predictors of patient outcomes. Furthermore, the study highlights the importance of considering demographic factors, such as gender, in interpreting these biomarkers, as variations were observed in inflammatory responses between male and female patients. This knowledge can refine clinical approaches and enhance individualized patient care. While our research contributes to the understanding of inflammatory markers in this context, the limitations regarding the study's duration and sample size emphasize the need for further investigation. Future studies should involve larger, diverse populations and multicenter collaborations to validate these findings and explore the potential for integrating these biomarkers into routine clinical practice. By fostering a more comprehensive approach to monitoring and managing head and neck abscesses, we aim to improve treatment outcomes and reduce the associated healthcare burden.
